# Atypical wound trajectory after a tangential pistol shot

**DOI:** 10.1007/s00414-022-02905-y

**Published:** 2022-11-10

**Authors:** Constantin Lux, Mattias Kettner, Jan M. Federspiel, Frank Ramsthaler, Marcel A. Verhoff

**Affiliations:** 1 Institute of Legal Medicine, University Hospital of Frankfurt, Goethe University, Kennedyallee 104, 60596 Frankfurt/Main, Germany; 2 Institute of Legal Medicine, University of Saarland, Building 49.1, 66421 Homburg/Saar, Germany

**Keywords:** Gunshot, Murder, Bullet trajectory deflection, Autopsy, Post-mortem CT

## Abstract

Three intermediate-range shots from a Browning, model 1955, 7.65 mm caliber, pistol were fired from the driver’s seat of a car at a woman in the passenger seat. She sustained three wounds: An, ultimately fatal, penetrating head wound, a graze wound across her forehead, and a tangential, perforating, wound, with bullet entry over the medial sternum and exit through the right flank. Neither postmortem CT nor forensic autopsy discovered bony thoracic injuries or perforations of the thoracoabdominal cavities. There was pulmonary contusion in the medial lobe of the right lung and hemorrhage in the adipose tissue around the right kidney. The tangential bullet had left an almost 40-cm-long wound channel through a pronounced layer of subcutaneous fat. Based on 3D reconstructed CT-data determinations, a straight bullet trajectory between entry and exit wounds would have traversed the abdominothoracic cavities, right lung, and liver. The actual trajectory, however, described a prominent curve, without signs of deflection by bone. Postulated explanations for this unusual bullet track are that the woman was twisting her body in a dynamic scene when the bullet struck; further, due to its shallow angle of incidence on the skin, the bullet was deflected to an intracutaneous path. Additionally, soft tissue resistance may have caused the bullet to yaw. Caution should, thus, be exercised when reconstructing bullet trajectories solely from entry and exist wounds, also for bullet wounds through basically homogenous soft tissues.

## Introduction

The documentation and interpretation of firearm injuries are routine in forensic casework. These tasks are performed not only for deceased victims in the context of autopsies, but also for living victims within the scope of clinical medicolegal examinations. In both situations, the macromorphological examinations are performed in conjunction with imaging methods [1–7]. In the reconstruction of shooting incidents, projectile trajectory and shot range are important aspects that need to be determined. Commonly, the primary goal of these reconstructions is to allow the inclusion or elimination of various scenarios during the shooting incident. In the case of perforating bullet wounds, the bullet trajectory can, for example, be calculated by simple trigonometry from the measurements for the specific locations of the entry and exit wounds on the body. This approach is, however, only applicable if the bullet followed an essentially straight path through the body and was not deflected along its way [8, 9]. When projectiles glance off anatomical structures such as bones or teeth, or when they tangentially pass through fluid-filled cavities, large deflections in trajectory are possible [8]. Generally, the magnitude of these deflections is influenced by the interaction of various factors, such as construction-related properties of the projectile, velocity and energy of the projectile as it enters the body, trajectory of the projectile, transfer of energy from the projectile to the penetrated material, length of the wound channel, and exact location where the projectile encounters body structures [8, 10–13].

In the following we report on an atypical wound trajectory through subcutaneous adipose tissue, in which the bullet followed a curved path, without being deflected by bone or organ structures.

## Case report

**Fig. 1:**
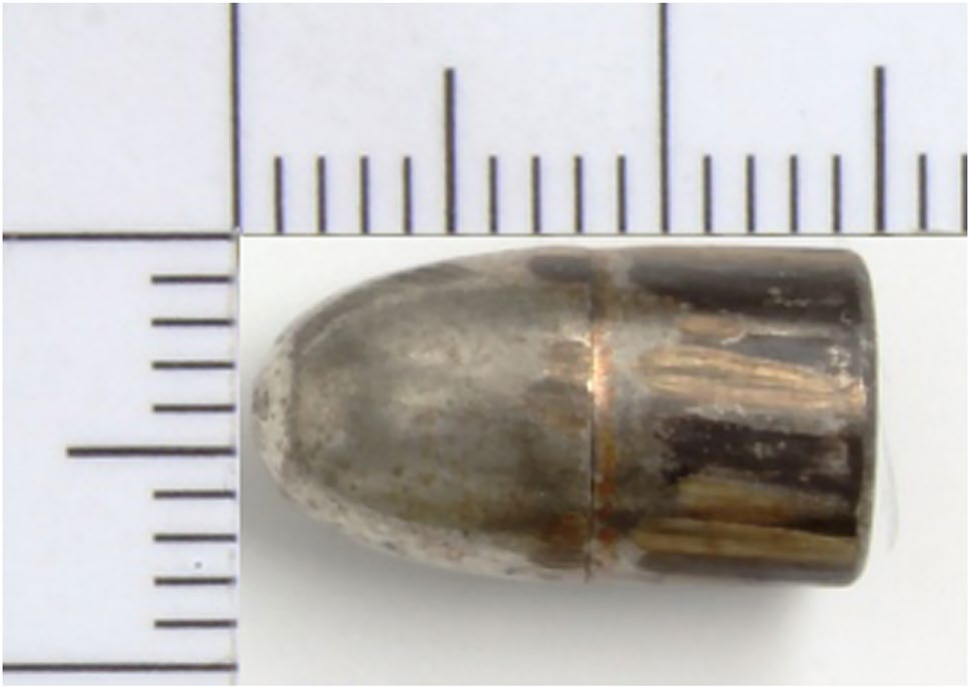
The bullet retrieved from the passenger door sill. Ultimately, this bullet could be attributed to the tangential shot. The bullet was undamaged, apart from the typical grooves left by the pistol barrel

A man in a private car brought a seriously injured woman to the emergency department of a hospital. Shortly after arrival, the woman had to be resuscitated; however, resuscitation was unsuccessful. Because the woman’s injuries were ostensibly bullet wounds, the hospital informed the police. When the police subsequently questioned the man who had brought her, who was still in the waiting room of the hospital, about the incident, he stated that he had shot his partner in the car during a quarrel. He had been sitting in the driver’s seat at the time and his partner in the passenger seat. Criminal police officers then recovered a Browning, model 1955, 7.65 mm caliber, pistol from the man’s car, as well as a basically undamaged lead core bullet from the sill of the passenger door (Fig. 1).

### Autopsy results

**Fig. 2:**
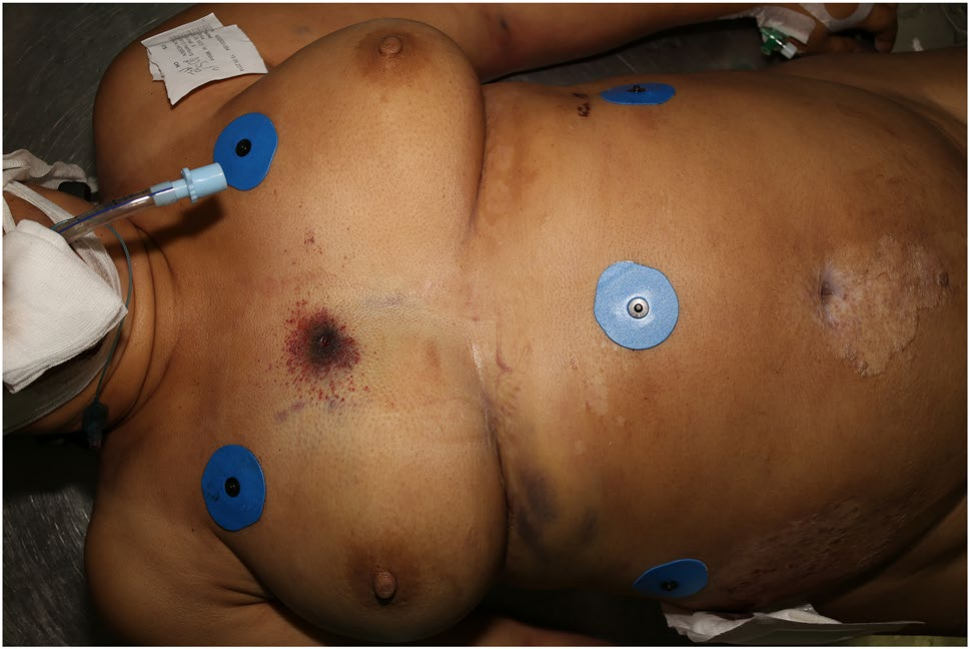
Entry wound from the tangential shot over the sternum, 2.5 cm to the right of the midline

**Fig. 3:**
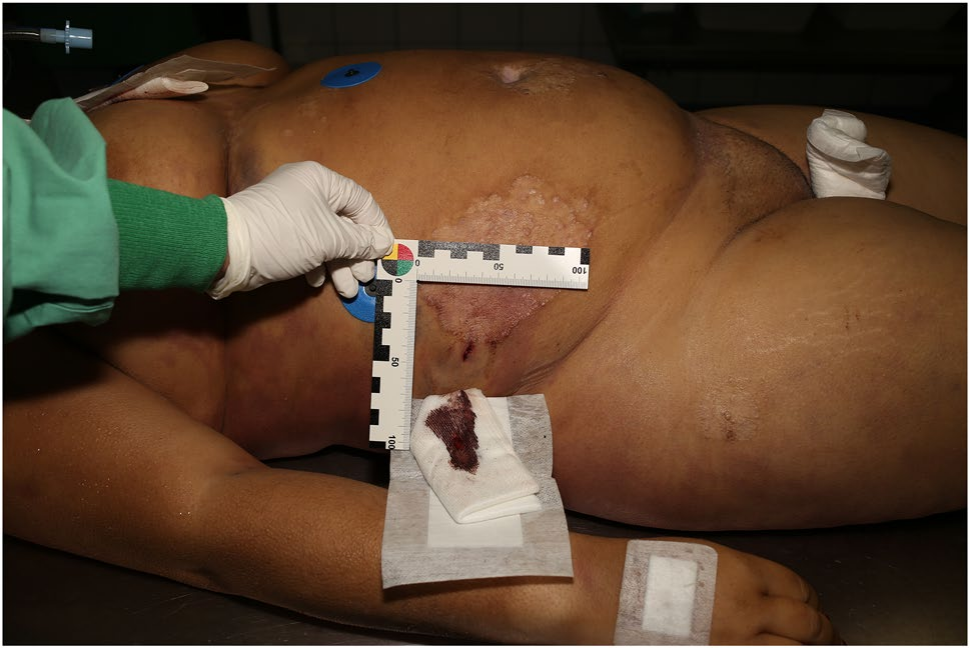
Exit wound on the right flank

The cadaver of the middle-aged woman weighed 84 kg and was 161 cm long. Three bullet wounds were found. Due to the deposition of gunshot residue, the shots were considered to have been fired from an intermediate range. There was a horizontal graze wound across her forehead, running from left to right. In addition, there was a penetrating bullet wound in the left parietal bone. This was the ultimately fatal injury. The third injury was a perforating bullet wound, with an entry wound over the sternum, 2.5 cm to the right of the midline and 126 cm from the bottom of the feet (Fig. 2). The exit wound was located in the right flank, 22 cm to the right of the midline and 98 cm from the bottom of the feet (Fig. 3).

**Fig. 4:**
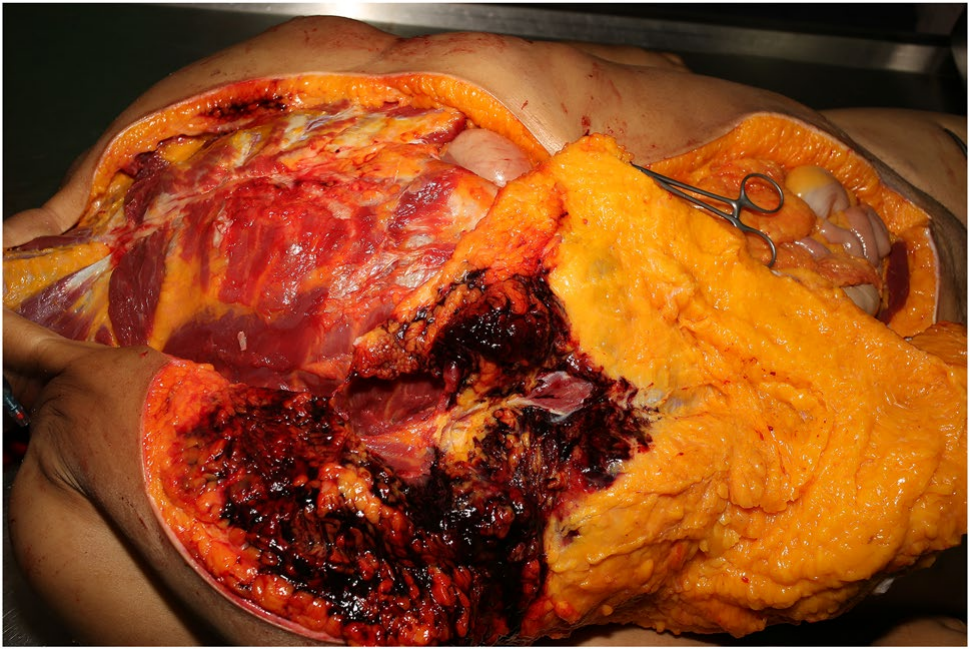
Dissection of the subcutaneous adipose tissue on the right, anterior side of the torso. The bullet had only injured subcutaneous adipose tissue

Post-mortem CT had shown that there were no bony injuries in the thorax and that neither the thoracic nor the abdominal cavities had been penetrated. Furthermore, there were no metallic particles as a sign of potential contact between bullet and bone. An increase in density had, however, been noticed in the medial lobe of the right lung. The CT findings were corroborated at autopsy, in which pulmonary contusion in the medial lobe of the right lung and further signs of hemorrhage in the adipose tissue around the right kidney were found. The wound channel from the perforating shot described an almost 40-cm-long, curved path through a well-developed layer of subcutaneous fat. The shot was, therefore, considered to be tangential (Fig. 4).

**Fig. 5:**
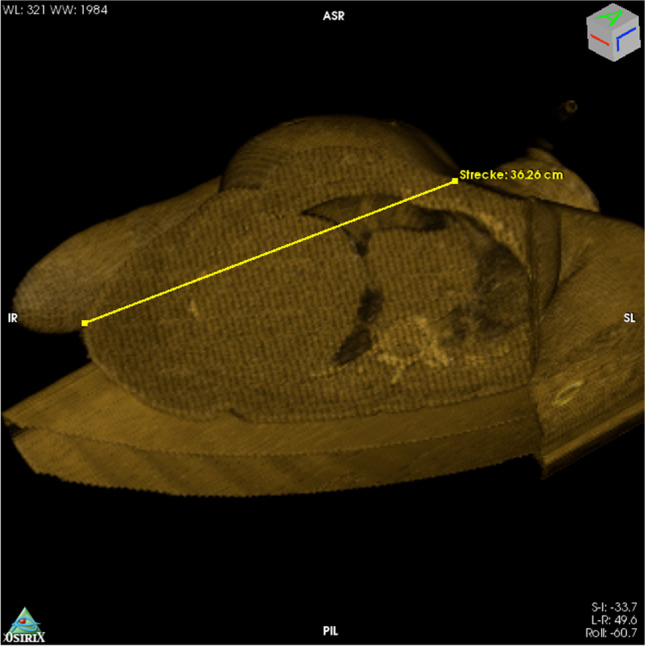
3D reconstructed CT datasets in volume-rendering mode, depicting the virtual wound trajectory and measurement of the straight connection between entry and exit wounds. After aligning the torso in the frontal plane, the cutting tool in OsiriX was used to cut the image along the bullet track. The parts of the torso that faced towards the lower left were eliminated. The ensuing cross section was then aligned in the frontal plane, and the exit and entry wounds were connected, using a measuring tool. The approximately 36-cm-long bullet trajectory can be seen to pass through the thoracic and the abdominal cavities

On the basis of the wound trajectory identified in 3D-reconstructed CT data, in which the bullet track was determined as a straight line from entry to exit wound (measured distance: 36 cm), the expectation had been that the bullet would have entered the thoracic and the abdominal cavities and that it would have perforated the right lung and the liver (Fig. 5).

**Fig. 6:**
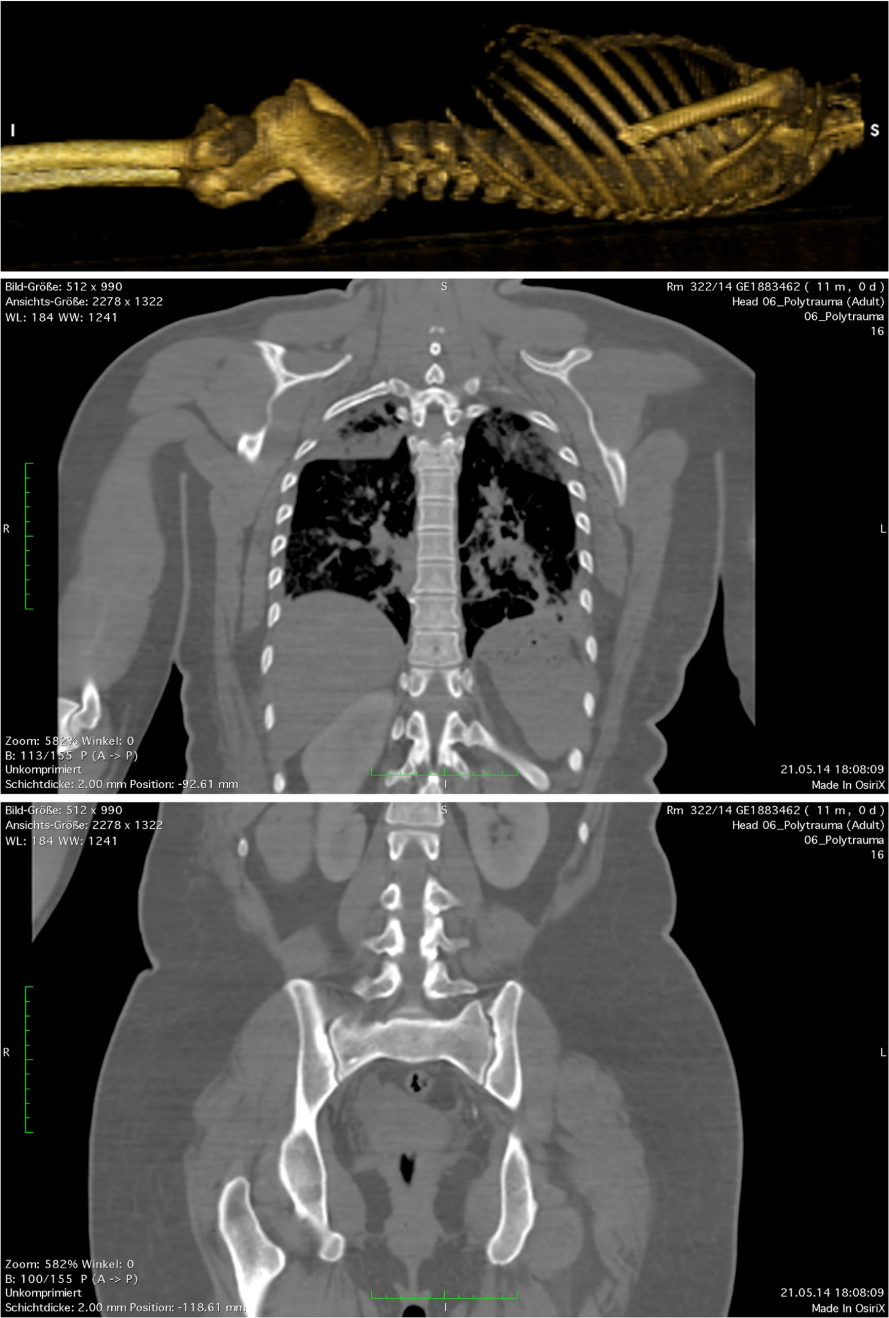
Top: Depiction of the skeletal torso after 3D reconstruction in volume-rendering mode (bone window). Middle and bottom: Cross sections reconstructed in frontal plane. In the top image, the kyphosis of the thoracic spine can be recognized; the middle image shows narrowing of the intervertebral spaces and formation of osteophytes; in the bottom image sacroiliitis can be recognized. The findings are consistent with ankylosing spondylitis

Further CT findings had been a pronounced thoracic kyphosis, narrowing of the intervertebral spaces and the formation of bony bridges between the vertebrae, mainly in the region of the thoracic spine, as well as signs of sacroiliitis (Fig. 6). These findings are consistent with ankylosing spondylitis.

### *Trial*

**Fig. 7:**
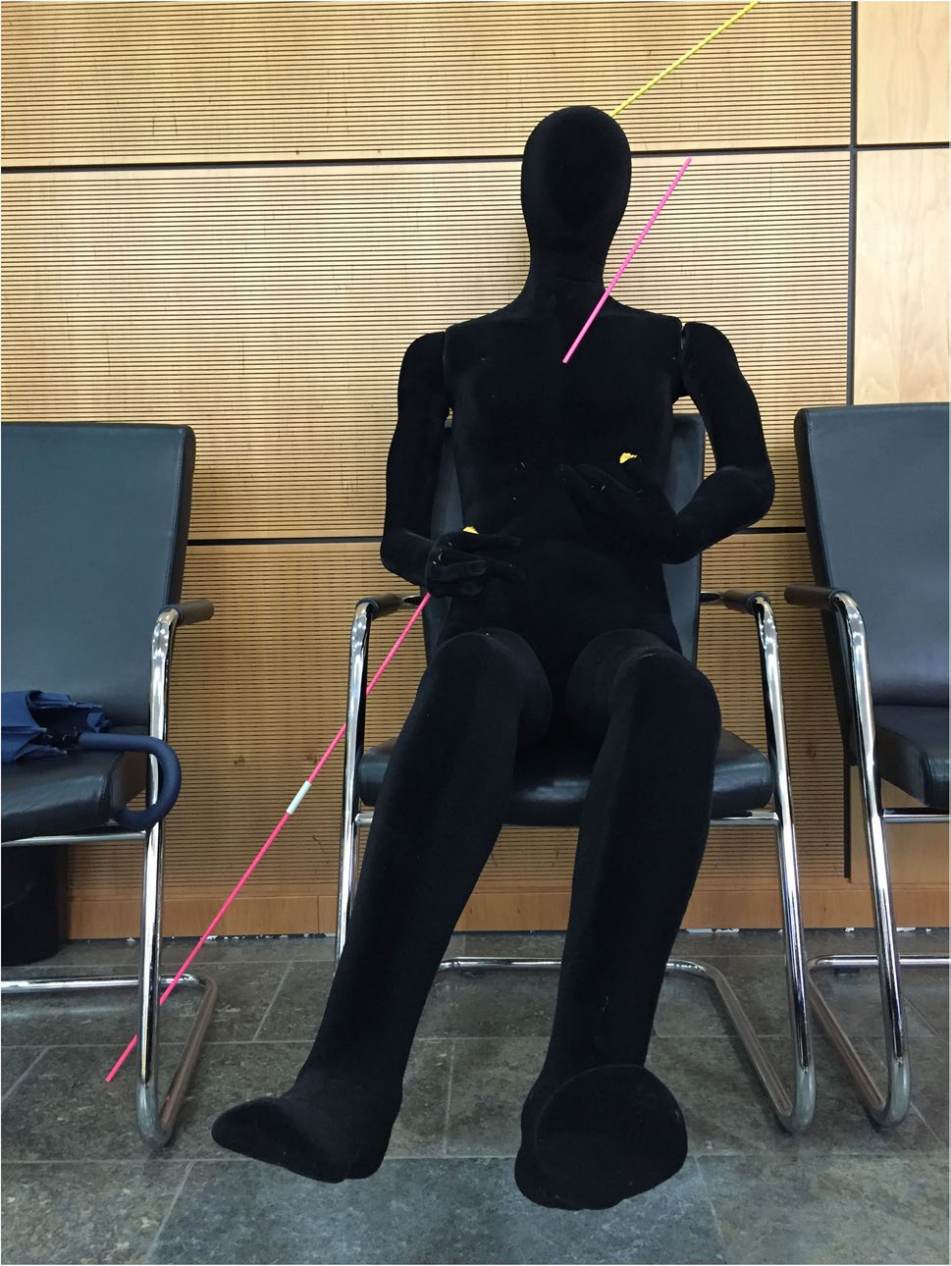
“Ballistic dummy” illustrating the reconstructed trajectories for the fatal penetrating bullet wound to the head (yellow) and the tangential shot (violet), in the court room

The following forensic findings were presented during the main hearing of the trial: All three pistol shots had been fired from a range of 3 to 30 cm. The bullet that had been found on the sill of the passenger door of the car could be identified as that from the tangential, perforating, shot. The bullet was undamaged, apart from the typical grooves left by the pistol barrel. In a joint demonstration, the ballistic and medicolegal expert witnesses used trajectory rods and a dummy that was only slightly larger, although less massive, than the victim to reconstruct the three pistol shots for the court (Fig. 7). The goal of this three-dimensional reconstruction was to illustrate the scene within the car graphically, in particular, to elucidate why the wound trajectory from the tangential shot could not be satisfactorily explained alone on the basis of the victim’s body position when the shot was fired, e.g., leaning forward with a twisted torso. A curved trajectory was, therefore, postulated for this bullet. In the case of this shot, an incidence angle of 35° in relation to the body’s longitudinal axis in the frontal plane had been calculated from the measured locations of the entry and exit wounds. While the bullets evoking the graze wound and the penetrating wound would have had to be fired with the pistol barrel oriented approximately in the frontal plane of the victim’s head, the tangential bullet would have, thus, likely been fired from a position anterior to this plane, as might have occurred if the victim on the passenger seat had twisted her upper body towards the shooter in the driver’s seat. During the trial, the expert witnesses, however, also cautioned that there were constraints on the accuracy of the calculated incidence angle due to the possibility that the bullet had been deflected along its path through the body. The actual incidence angle of the bullet in respect to the longitudinal axis of the victim’s body might thus have been greater than calculated; similarly, the position of the pistol might have been lower. Furthermore, at the time the shot was fired, the pistol could also have been oriented closer to the frontal plane than assumed, as in the two other shots.

## Discussion

In the reported case, a woman sustained three intermediate-range shot wounds after being fired at from the driver’s seat on the left side of a car. From a medicolegal perspective, the bullet trajectories for a graze wound on the victim’s forehead and the fatal penetrating gunshot wound to the head were simple to reconstruct.

The wound track left by the third, tangential, bullet was, however, so unusual that we believe it merits reporting. In the trajectory analysis of this wound from 3D reconstructed CT data sets, the linear distance measured between entry and exit wounds was approximately 36 cm. In this straight trajectory through the body, the bullet would have had to traverse the thoracic and abdominal cavities; however, the actual wound channel was not found to pass through either of these cavities. The bullet had, instead, followed an approximately 40-cm-long, nonlinear trajectory though the subcutaneous adipose tissue. Contact with bone could be excluded as a reason for bullet deflection. The contusion in the medial lobe of the right lung and the hemorrhage in the adipose tissue around the right kidney were, therefore, likely caused by expansion of the temporary cavity around the wound channel.

In an attempt to explain this atypical wound trajectory, possible body positions of the victim, in addition to ribcage positions while breathing, were considered, such as that she may have twisted her upper body, or have leaned forward or slumped sideways when the shot was fired. To aid this elucidation attempt, a dummy was used during the main trial to explore all conceivable positions of the victim’s body at the moment the shot was fired. Even when possible contributing effects such as tissue compression while twisting—and hence local increase in tissue density or rigidity—were additionally taken into account, none of the explored body positions could satisfactorily explain the unusual trajectory. Moreover, due to the kyphosis and intervertebral ossification that were noted in the postmortem CT, the mobility of the victim’s spine was manifestly restricted. This circumstance also had to be factored into the trajectory analysis. In the CT scan, it could, moreover, be seen that the victim’s upper body remained noticeably bent forward even when the body lay in a supine (post-mortem) position. The virtual trajectory between entry and exit wounds would, hence, have passed through her thoracic and abdominal cavities also in this position.

After consideration of all circumstances, the only viable explanation for the atypical trajectory of the bullet through the body is to postulate that it was deflected from its straight path to a curved trajectory, without glancing off bone. This hypothesis is strengthened by the observation that the only visible alterations on the perforating bullet were similar to those that would be expected for a bullet from the same pistol that had been fired into gelatin. In all other respects, the bullet was undamaged. The findings in our case report also tie in with the reported results from experimental studies in which significant deflections in trajectory were found for bullets passing through homogenous soft-tissue simulants [11, 12]. In the case of projectiles from long-barreled firearms, this phenomenon may be explained by tipping or yawing of the bullet along its track through soft tissues. The resulting asymmetrical distribution of pressure on the bullet nose would exert a lateral force on the bullet, which would cause lateral acceleration and, thus, deflection of the projectile from a straight path on its way through the tissue [14]. Although this effect would be expected to be significantly less pronounced for short-barreled firearms, due to constructional properties, deflections of more than 6° for bullet tracks longer than 20 cm have been reported in the literature, depending on the combination of projectile and weapon [11].

A further explanation that could be postulated for the atypical wound trajectory in our case is that the bullet may, after tearing through the skin (entry wound) and passing through the subcutaneous adipose tissue for a few centimeters, have traveled towards the skin again. Due to the shallow incidence angle, the bullet may then have failed to penetrate the dermis from below and exit the body. Because of the skin’s elasticity, the bullet may then have been forced to follow a path parallel to the dermis until it reached a point, on the medial axillary line, where the radius of the skin’s curvature decreased far enough to increase the bullet’s angle of incidence to the extent that it allowed the bullet to penetrate the skin and exit the body.

In all, the case we report here further exemplifies the necessity of exercising due caution in identifying bullet trajectories solely on the basis of the locations of entry and exit wounds, even for wounds through essentially homogenous soft-tissues. Where the circumstances permit, it may, therefore, be more expedient to identify and use deflection points on bone, or perforations in serous membranes, along the first 10 cm of the wound channel, instead of the exit wound, to calculate the bullet trajectory [11]. Moreover, if CT data are available for the virtual identification of the wound trajectory, the difference between the supine position of the victim’s body during the CT scan and the victim’s actual position at the time the bullet was fired should be taken into account as a possible source of error in calculating the bullet trajectory [15].
